# Spatio-Temporal History of HIV-1 CRF35_AD in Afghanistan and Iran

**DOI:** 10.1371/journal.pone.0156499

**Published:** 2016-06-09

**Authors:** Sana Eybpoosh, Abbas Bahrampour, Mohammad Karamouzian, Kayhan Azadmanesh, Fatemeh Jahanbakhsh, Ehsan Mostafavi, Farzaneh Zolala, Ali Akbar Haghdoost

**Affiliations:** 1 Regional Knowledge Hub, and WHO Collaborating Centre for HIV Surveillance, Institute for Futures Studies in Health, Kerman University of Medical Sciences, Kerman, Iran; 2 Modeling in Health Research Centre, Institute for Futures Studies in Health, Kerman University of Medical Sciences, Kerman, Iran; 3 School of Population and Public Health, Faculty of Medicine, University of British Colombia, Vancouver, BC, Canada; 4 Virology Department, Pasteur Institute of Iran, Tehran, Iran; 5 Epidemiology Department, Pasteur Institute of Iran, Tehran, Iran; 6 Emerging and Reemerging Infectious Diseases Research Centre, Pasteur Institute of Iran, Akanlu, Kabudar Ahang, Hamadan, Iran; Centro de Biología Molecular Severo Ochoa (CSIC-UAM), SPAIN

## Abstract

HIV-1 Circulating Recombinant Form 35_AD (CRF35_AD) has an important position in the epidemiological profile of Afghanistan and Iran. Despite the presence of this clade in Afghanistan and Iran for over a decade, our understanding of its origin and dissemination patterns is limited. In this study, we performed a Bayesian phylogeographic analysis to reconstruct the spatio-temporal dispersion pattern of this clade using eligible CRF35_AD *gag* and *pol* sequences available in the Los Alamos HIV database (432 sequences available from Iran, 16 sequences available from Afghanistan, and a single CRF35_AD-like *pol* sequence available from USA). Bayesian Markov Chain Monte Carlo algorithm was implemented in BEAST v1.8.1. Between-country dispersion rates were tested with Bayesian stochastic search variable selection method and were considered significant where Bayes factor values were greater than three. The findings suggested that CRF35_AD sequences were genetically similar to parental sequences from Kenya and Uganda, and to a set of subtype A1 sequences available from Afghan refugees living in Pakistan. Our results also showed that across all phylogenies, Afghan and Iranian CRF35_AD sequences formed a monophyletic cluster (posterior clade credibility> 0.7). The divergence date of this cluster was estimated to be between 1990 and 1992. Within this cluster, a bidirectional dispersion of the virus was observed across Afghanistan and Iran. We could not clearly identify if Afghanistan or Iran first established or received this epidemic, as the root location of this cluster could not be robustly estimated. Three CRF35_AD sequences from Afghan refugees living in Pakistan nested among Afghan and Iranian CRF35_AD branches. However, the CRF35_AD-like sequence available from USA diverged independently from Kenyan subtype A1 sequences, suggesting it not to be a true CRF35_AD lineage. Potential factors contributing to viral exchange between Afghanistan and Iran could be injection drug networks and mass migration of Afghan refugees and labours to Iran, which calls for extensive preventive efforts.

## Introduction

Human immunodeficiency virus type 1 (HIV-1), is a highly mutating RNA virus [1]. HIV-1 group M, the pandemic branch of HIV, is reported to have originated from western-central Africa in around 1900–1930 and then started to spread around the world from the 1950s [2, 3]. During its evolutionary history, the genetic variability of the virus has led HIV-1 group M to derive different subtypes (A–D, F–H, J, and K), sub-subtypes (A1–A4, and F1–F2), and recombinant forms [4].

HIV-1 Circulating Recombinant Form 35_AD (CRF35_AD), a group M recombinant, is the result of subtype A1 and subtype D recombination. Molecular epidemiological studies suggest the predominance of HIV-1 CRF35_AD in both Afghanistan and Iran [5–12]. Data on the Los Alamos HIV database show that of the total number of HIV-1 sequences available from Afghanistan (n = 26) and Iran (n = 974), respectively 756 (78%) and 16 (67%) sequences are of CRF35_AD classification [4]. Other than Afghanistan and Iran, CRF35_AD was only reported from three Afghan refugees living in Pakistan (in 2009) [13]; however, the clade has not yet been reported among Native Pakistanis. Lastly, a CRF35_AD-like *pol* sequence, is reported from a 36-year-old woman living in USA (in 2010), with unknown source of infection [14].

Despite the predominance of HIV-1 CRF35-AD in Afghanistan and Iran for more than a decade, our knowledge is scarce about the onset date of this epidemic in these countries and the spatio-temporal dispersion pattern of the virus across both countries. Some hypotheses, however, have been made in this regard, proposing a unidirectional dispersion of the CRF35_AD across Afghanistan and Iran [8, 10, 11, 13]. But, these hypotheses have not been systematically investigated in a phylogeographic framework. Moreover, it is not known if the CRF35_AD strains reported from USA and Afghan refugees living in Pakistan are epidemiologically linked to the CRF35_AD epidemic in Afghanistan and Iran.

Of parental subtypes of the CRF35_AD clade, subtype A1 is observed in most countries in the region. Parental subtype D, however, is not observed in countries neighboring Afghanistan and Iran (except in Saudi Arabia) [4]. This clade circulates in most countries of East Africa, including Uganda, Sudan, Somali, Tanzania, and Djibouti [4, 15]. Potential linkages between parental subtypes circulating in the region or the rest of the world, and the CRF35_AD epidemic in Afghanistan and Iran are unclear.

Given the knowledge gap about the epidemic history and dissemination pattern of the HIV-1 CRF35_AD, we conducted the present study to: (i) reconstruct the spatio-temporal history of the CRF35_AD in Afghanistan and Iran; (ii) investigate if the CRF35_AD strains available from USA and Afghan refugees living in Pakistan link to the CRF35_AD epidemic in Afghanistan and Iran; and (iii) explore genetic relationships between CRF35_AD sequences and parental A1 and D strains available worldwide.

## Methods

### Ethics statement

The Ethics Committee of Kerman University of Medical Sciences reviewed and approved the study protocol (Ethical Code: IR.KMU.REC.1394.430). All alignments are accessible to the readers upon request.

### HIV-1 CRF35_AD sequence datasets

All HIV-1 CRF35_AD *gag* and *pol* sequences from Afghanistan and Iran, with known sampling date, were downloaded from the Los Alamos HIV Database by March 2016 [4]. In Afghanistan, this included 16 CRF35_AD sequences retrieved from two metropolitan cities in the East (Kabul) and West (Herat) of Afghanistan (n = 13), as well as a community of Afghan refugees living in Pakistan (n = 3). From Iran, 430 CRF35_AD sequences were retrieved from six cities in the North East (Mashhad), West (Borujerd, Sanandaj, and Kermanshah), South (Shiraz) and Center (Tehran). One sequence was also reported by Pyne, et al. (2013) as CRF35_AD, although not characterized at full-length. This sample has been obtained from a 36-year-old woman with unknown nationality living in Virginia State (USA) [14]. This sequence was included in our phylogeographic analyses to check for its possible epidemiological linkage to CRF35_AD_Afghan-Iranian_ sequences. As the single sequence could not be representative of the epidemic status in USA, however, no state- or country-level inference was made based on this sequence.

**Fig 1:**
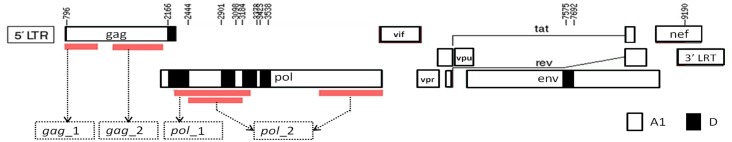
Genome structure of HIV-1 CRF35_AD. In this recombinant lineage, subtype D fragments are found within the *gag*, *pol*, and *env* genomic regions, in a genomic background that matches subtype A1. We created four non-overlapping alignments, each of which belonged to one parental lineage of the CRF35_AD only. These alignments include *gag*_1, *gag*_2, *pol*_1, and *pol*_2. From the *pol*_1 alignment, which mainly covers parental D fragments, the A1 sections were removed, and the remaining D sections were concatenated. A similar approach was taken to remove parental D section from the *pol*_2 alignment, which mainly covers parental A1 fragments. Details of nucleotide positions related to these alignments can be found in the Methods.

Analyses were performed on four non-overlapping alignments, each covering partial *gag* or partial *pol* genes and belonging only to one parental lineage of the CRF35_AD (i.e., subtypes A1 or D, see Fig 1). This was done to make phylogeographic inferences based on multiple loci and bring both parental subtypes of the CRF35_AD into the analyses (see below and Fig 1). From the *gag* gene, two alignments were obtained naming *gag_*1 and *gag*_2, which belonged to parental subtype A1. The *gag*_1 alignment covered entire p17 and partial p24 regions (HXB2 numbering: 790–1230; 440 bp; n = 64). The *gag*_2 alignment covered partial p24 and entire p2/p1 regions (HXB2 numbering: 1460–2130; 670 bp; n = 38). From the *pol* gene also, two alignments were obtained naming *pol*_1 and *pol*_2, which belonged to parental subtype D and subtype A1, respectively. The *pol*_1 alignment covered partial protease and reverse transcriptase (PR/RT) regions (HXB2 numbering: 2252–3270; 1018 bp; n = 115). The *pol*_2 alignment covered partial PR/RT and complete integrase (INT) regions (HXB2 numberings: 2445–3184 and 4230–5093, respectively; 1602 bp; n = 270). Two A1 fragments were removed from the *pol*_1 alignment (HXB2 numberings: 2444–2901 and 3098–3184, respectively), and the remaining parental D segments were concatenated. This led to a final fragment of 475 bp at the *pol*_1 region. A similar approach was applied to remove the D segment from the *pol*_2 alignment (HXB2 numbering: 2901–3098) and to achieve a pure A1 fragment. This led to a final fragment of 1405 bp at the *pol*_2 region. These alignments are called HIV-1_CRF35_AD_ (Fig 1, S1 Table).

### Parental subtype A1 and subtype D sequence datasets

To find parental subtypes that are genetically close to the CRF35_AD strains, we generated four extra alignments, and added parental A1 (in the *gag*_1, *gag*_2, and *pol*_2 regions) or D (in the *pol*_1 region) sequences to the HIV-1_CRF35_AD_ datasets. A preliminary Maximum Likelihood (ML) phylogenetic analysis of these datasets was performed using MEGA v.6 software [16], with the GTR+G4+I model of nucleotide substitutions. The results indicated that among all parental sequences available worldwide, CRF35_AD sequences were genetically similar to parental sequences from Kenya, Uganda, and a cluster containing Afghan refugees living in Pakistan and Native Pakistanis (S1 Fig). Therefore, only parental strains from these locations were retained and included in the subsequent Bayesian phylogeographic analyses (S2 Table). This approach was taken to reduce the number of discrete locations in the transition matrix. At this step, in order to reduce the chance of losing similar parental strains in the ML phylogenetic analyses, a low bootstrap threshold (50%) was considered [17, 18]. In the Bayesian phylogeographic analyses, however, a higher threshold was set to decide about the genetically similar parental strains (i.e., Posterior Clade Credibility >0.7. See below and S2 Fig).

### Sequence alignment and analysis of phylogenetic signal

Sequences were aligned using the Clustal W algorithm as implemented in MEGA v.6 software [16]. Substitution saturation analyses were performed in the DAMBE v.5.5.2 program [19] using GTR nucleotide substitution model for estimation of pairwise genetic distances. To investigate the tree likeliness of each alignment, likelihood mapping analyses were performed in the Tree-Puzzle v.5.2 program [20, 21]. A gamma model of rate heterogeneity with eight categories (G8) was assumed, and the number of random quartets was set to 10^4^.

### Genetic characterization

Subtype assignment of all *gag* and *pol* sequences was confirmed by maximum likelihood phylogenetic analyses using MEGA v.6 software [16]. To confirm the CRF35_AD structure of Iranian and Afghan full-length sequences (n = 22), similarity plotting was performed in Simplot v.3.5.1 software [22] with a window and step size of 300 and 20 nucleotides, respectively. HIV-1 reference sequences were obtained from the Los Alamos HIV Database [4]. Sequences were excluded if they showed evidence of incorrect subtype assignment, frameshift mutations, or stop codon positions. Only one sequence per patient was included. The *pol* sequences from therapy-naive subjects and/or with no major drug resistance mutations were selected to avoid potential biases due to convergent evolution caused by antiretroviral therapy.

### Phylogenetic analysis

ML trees were reconstructed under the GTR+G4+I model of nucleotide substitutions, which was selected using the jModeltest v.0.1.1 program [23]. ML trees were reconstructed using MEGA v.6 software [16]. Heuristic tree search was performed using the Subtree-Pruning-Regrafting branch-swapping algorithm [24]. Reliability of the obtained tree topologies was estimated with Bootstrap testing (1000 replicates). Final trees were visualized in the FigTree v.1.4.2 program [25].

### Spatio-temporal dispersion patterns of CRF35_AD

The age of the most recent common ancestor (*T*_*mrca*_, years) and the spatio-temporal dispersion pattern of CRF35_AD strains were estimated using a Bayesian Markov Chain Monte Carlo (MCMC) approach in BEAST v.1.8.1 software [26]. BEAGLE library was used to improve the computational speed [27]. Although the GTR was the model proposed by jModeltest program [23], summarizing this model to the HKY did not alter the parameters estimates yet resulted in tighter 95% Bayesian Credible Intervals (BCI) and improved chain mixing. Therefore, phylogeographic analyses were performed under the HKY+G4+I nucleotide substitution model. We also used uncorrelated lognormal relaxed clock model [24], the best fitting model across all genomic regions, and Bayesian Skyline coalescent tree prior [28] in our phylogeographic analyses.

As CRF35_AD sequences were not sampled over a sufficiently wide range of dates (see S1 Table), they could not be utilized for direct evolutionary rate estimation. Therefore, rates of evolution for different genomic regions were estimated from pure subtype A1 and D sequences spanning 28 (in *gag*_1, *gag*_2, and *pol*_2 alignments) and 26 (in *pol*_1 alignment) years, respectively. In these analyses, previously estimated rates for the *gag (*1.5–6.1 × 10^−3^) [29–32] and *pol* (1.0–5.6 × 10^−3^) [29, 31–38] regions of the HIV-1 group M were used as uniform priors. The estimated evolutionary rates were then incorporated as fixed prior intervals when analyzing CRF35_AD sequences.

Migration events of CRF35_AD were inferred using discrete non-reversible diffusion models [39] and Bayesian Stochastic Search Variable Selection (BSSVS) approach [39]. We performed our phylogeographic analyses treating each city as a discrete state to provide a fine-scale representation of the spatio-temporal dynamics of the virus. However, to reduce the size of the transition matrix, only between-country viral migration rates were tested and reported. The BSSVS approach was implemented in the SPREAD v.1.0.7 program [40] using a cut-off value of three for the Bayes Factor (BF) test. Significant non-zero rates obtained by the BSSVS approach were visualized in Google Earth (http://earth.google.com).

Two independent MCMC chains were run for 50–500 million generations. The runs were then combined using the LogCombiner v.1.8.1 program [41]. Convergence of each parameter was visually evaluated in Tracer v.1.6 [42], after burning the initial 20% of the samples. The Maximum Clade Credibility (MCC) tree was constructed in the TreeAnnotator v.1.8.1 program [43] and visualized using the FigTree v.1.4.2 program [25]. Nodes with Posterior State Probability (PSP) and Posterior Clade Credibility (PCC) of 0.7 or greater were considered as statistically reliable. To infer about potential contributing factors in viral dispersion, socio-behavioural variables, risk groups, and travel history of CRF35_AD-infected individuals were retrieved from the Los Alamos HIV database [4] and the literature [6–10, 12, 13, 44–50] (see S4 Table).

### Sensitivity analysis

As most available CRF35_AD sequences were from Iran (n = 432) rather than Afghanistan (n = 16), we generated a set of more “balanced” datasets containing up to 19 Iranian CRF35_AD strains at each genomic region (S3 Table). This approach was taken to investigate the sensitivity of our key parameters estimates to the data. These parameters included the tree topology and height, the *T*_*mrca*_ of the CRF35_AD_Afghan-Iranian_ cluster, dispersion pattern of the virus between Afghanistan and Iran, and the evolutionary history of the CRF35_AD-like strain available from USA. Model and prior setting for balanced analyses were similar to the analyses performed on the “complete” datasets.

### Sequences

All HIV-1 sequences analyzed in this study have been deposited in GenBank under the accession numbers listed in S4 Table. With respect to sequences excluded due to drug resistance mutations, accession numbers are listed in S4 Table and their respective numbers are available in S1 and S2 Tables.

## Results

The substitution saturation and likelihood-mapping analyses showed that our datasets contained enough evolutionary information for reliable phylogenetic inferences (S3 and S4 Figs, respectively). Frameshift mutations or stop-codon positions were not observed in the data. CRF35_AD sequences branched in a highly supported monophyletic cluster (bootstrap value> 90%) that contained CRF35_AD reference sequences, confirming their original classification (S5 Fig). The North American CRF35_AD-like sequence branched with this cluster with a Bootstrap of 78% (S5d Fig). Full-length CRF35_AD sequences displayed the same A1/D mosaic structure as the CRF35_AD reference sequences (S5 Fig).

Mean evolutionary rate of the *gag*_1, *gag*_2, *pol*_1, and *pol*_2 parental datasets were estimated at 4.1, 2.3, 4.4, and 2.9 × 10^−3^ substitutions/site/year, respectively. The coefficient of variation of the evolutionary rates deviated significantly from zero across all alignments, showing the fitness of the relaxed clock assumption (Table 1).

**Table 1:** Evolutionary parameters and their 95% BCIs.

	Evolutionary rate ^£^	Coefficient of variation		*T*_*mrca*_
	Mean (95% BCI)	Mean	95% BCI	Mean (95% BCI)
***gag*_1**	4.1 (3.2–5.1)	0.2	0.04–0.3	1990 (1987–1993)
***gag*_2**	2.3 (2.1–2.5)	0.6	0.4–0.8	1992 (1988–1994)
***pol*_1** ᵵ	4.4 (3.8–5.1)	0.6	0.5–0.7	1991 (1988–1992)
***pol*_2**	2.9 (2.4–3.4)	0.6	0.1–0.9	1990 (1987–1994)

^£^ Evolutionary rates are estimated from parental A1 and D datasets. These estimates are incorporated as fixed prior intervals for analysis of CRF35_AD sequences.

^ᵵ^ Only this dataset covered parental subtype Dof the CRF35_AD clade. ***T***_**mrca:**_ Time of the MRCA of the CRF35_AD strains circulating in Afghanistan and Iran.

**BCI:** Bayesian Credible Interval

**Fig 2:**
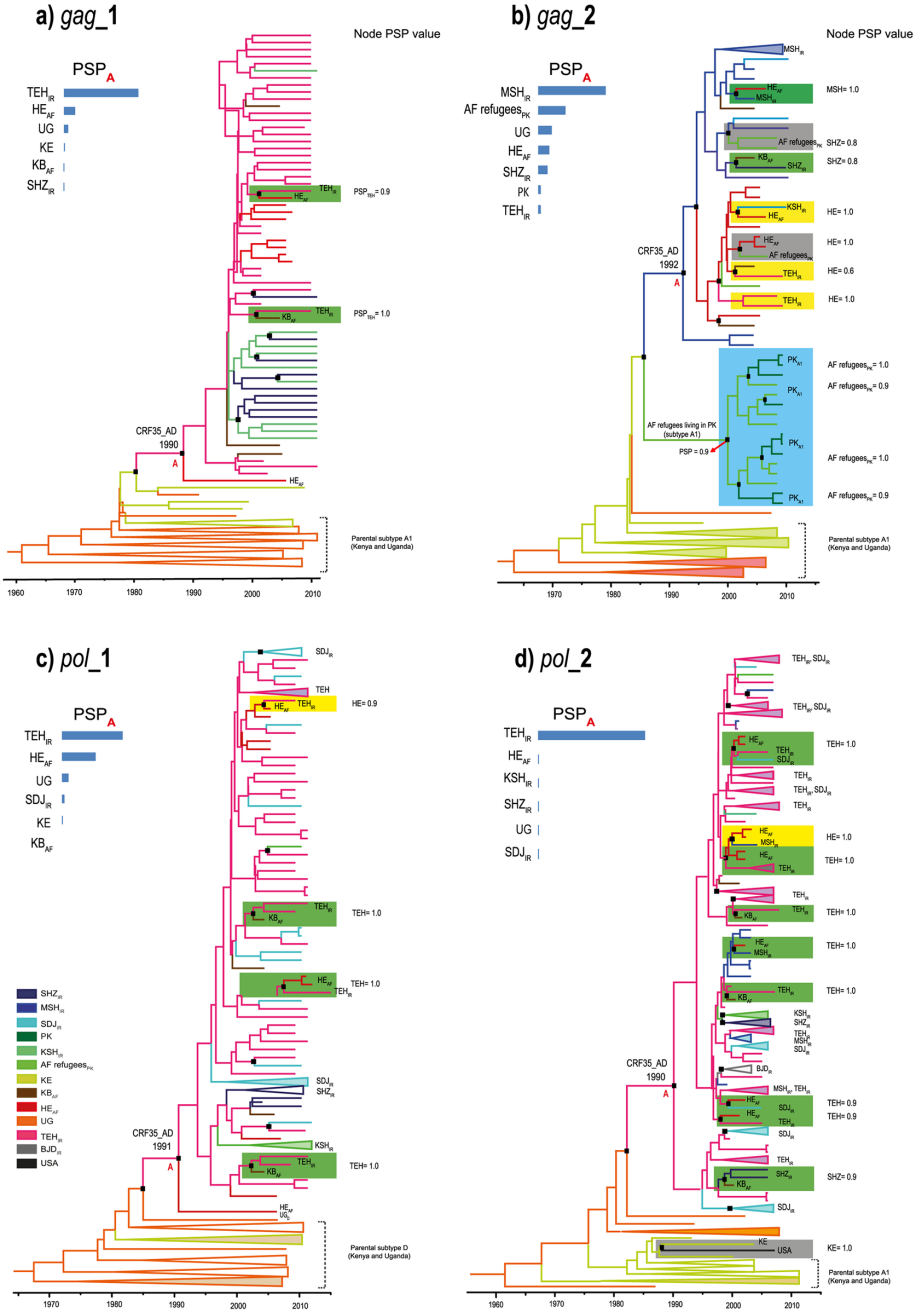
Time-scaled Bayesian MCC trees of the HIV-1 CRF35_AD. **(a) *gag*_1; (b) *gag*_2; (c) *pol*_1; (d) *pol_2*.** Key nodes with a posterior clade credibility of 0.7 or greater are marked with black rectangles. Most probable location state and its Posterior State Probability (PSP) are indicated in front of the nodes, corresponding to between-country migration events of CRF35_AD. These nodes are highlighted in Yellow (for Iran → Afghanistan transmission), green (for Afghanistan → Iran transmission), and gray (for Kenya → USA transmission). A highly supported subtype A1 cluster indicating transmission from Afghan refugees to Native Pakistanis is highlighted in blue (Part b). Other parental A1 or D sequences are collapsed for visual clarity. To the upper left of all phylogenies is a bar chart indicating posterior probabilities for the location states of the root of the CRF35_AD_Afghan-Iranian_ cluster (node A). The color code is indicated in the legend, on the lower left corner. **PSP:** Posterior State Probability; **A1:** HIV-1 subtype A1; **D:** HIV-1 subtype D; **AF:** Afghanistan; **IR:** Iran; **AF refugees**_**PK**_**:** Afghan refugees living in Pakistan; **BJD:** Boroujerd; **HE:** Herat; **KB:** Kabul; **KE:** Kenya; **KSH:** Kermanshah; **MSH:** Mashhad; **PK:** Pakistan; **SDJ:** Sanandaj; **SHZ:** Shiraz; **TEH:** Tehran; **UG:** Uganda.

Across all MCC trees, CRF35_AD_Afghan-Iranian_ sequences formed a well-supported monophyletic cluster (PCC> 0.7, named as CRF35_AD_Afghan-Iranian_ cluster onward). As shown in Table 1 and Fig 2, the *T*_*mrca*_ of this cluster was between 1990 and 1992 (lowest lower limit and highest upper limit of the 95% BCIs were 1987 and 1994, respectively). The choice of different genomic regions appeared to have no effect on the estimated dates (Table 1, Fig 2). The *T*_*mrca*_ of the Ugandan subtype A1 and D strains was estimated at around 1960 and 1970, respectively (Fig 2).

**Fig 3:**
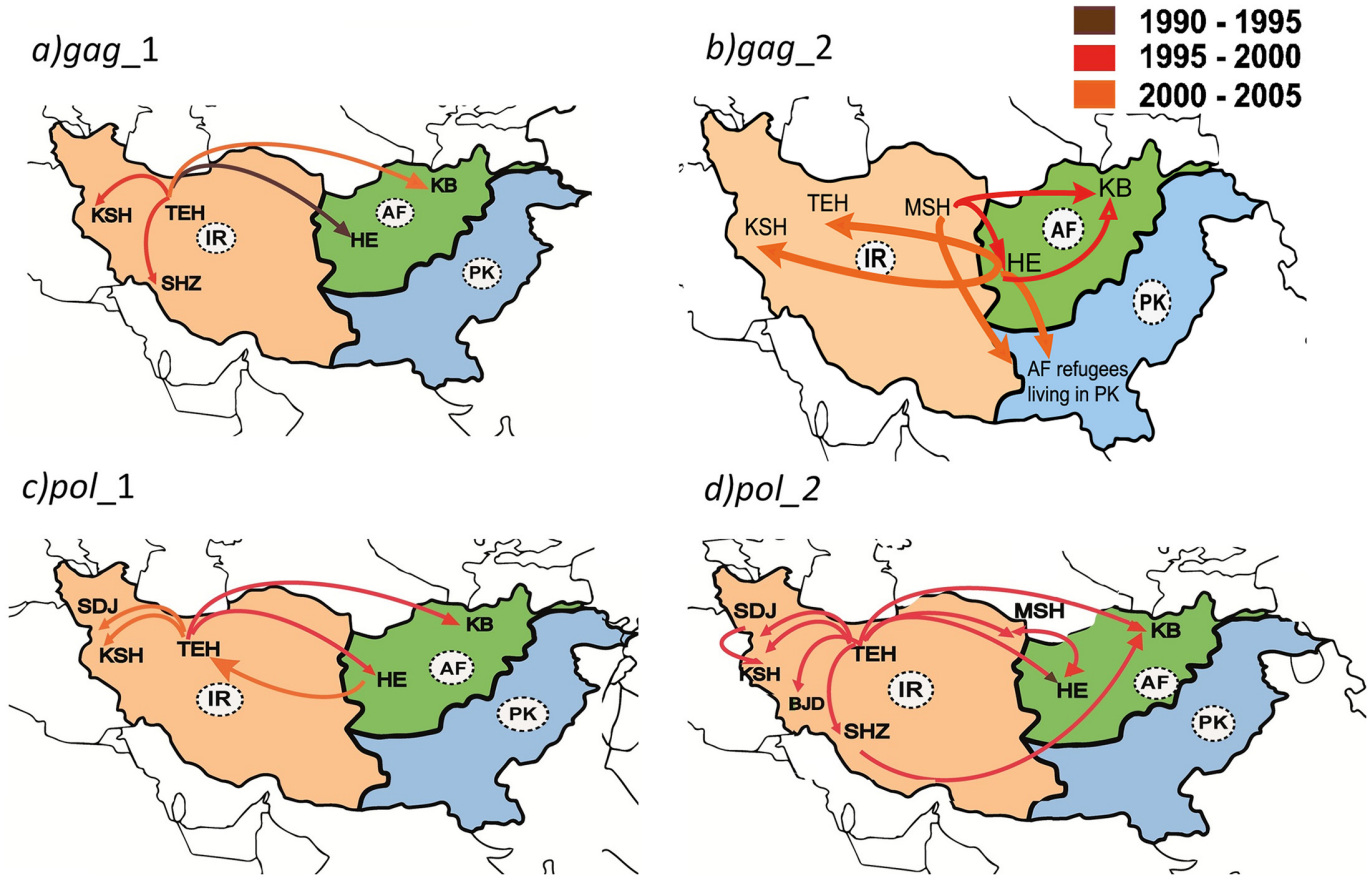
Spatio-temporal dynamics of HIV-1 CRF35_AD dispersion among Afghanistan, Iran, and Afghan refugees living in Pakistan, between 1990 and 2005. Arrows between locations represent branches in the time-scaled Bayesian MCC tree for the *gag*_1 **(a)**, *gag*_2 **(b)**, *pol*_1 **(c)**, and *pol*_2 **(d)** datasets. Only migration events supported by a posterior clade credibility of 0.7 or greater are indicated. Arrow colors reflect the timing of location transitions according to the legend at upper right corner. Lighter colors represent more recent transitions, and darker colors represent older history. **AF**: Afghanistan; **IR**: Iran; **PK**: Pakistan; **BJD**: Borujerd; **HE**: Herat; **KB**: Kabul; **KSH**: Kermanshah; **MSH**: Mashhad; **SDJ**: Sanandaj; **SHZ**: Shiraz; **TEH**: Tehran.

Within the CRF35_AD_Afghan-Iranian_ cluster, multiple CRF35_AD migration events were observed from Iran to Afghanistan and vice versa, suggesting a bidirectional dispersion pattern of the virus between both countries (Fig 2). Viral dispersion rates either from Afghanistan to Iran or vice versa were approximately one km per year and were statistically significant across all genomic regions (BF> 3). Our analyses also showed several independent CRF35_AD migration events between investigated locations within each country (Figs 2 and 3), suggesting that the virus has probably circulated within and between these countries. Three CRF35_AD sequences available from a community of Afghan refugees living in Pakistan also nested among Iranian and Native-Afghan CRF35_AD sequences. Nonetheless, the CRF35_AD-like *pol* sequence available from USA, showed an independent evolutionary history of other CRF35_AD sequences and diverged directly from Kenyan A1 strains in around 1986 (Fig 2d).

Consistent with the ML trees, the MCC trees also suggested a genetic relationship between CRF35_AD_Afghan-Iranian_ sequences and parental sequences from Kenya (Fig 2a), Uganda (Fig 2c and 2d), and a highly supported (PCC = 1.0) monophyletic cluster of A1 sequences containing Afghan refugees living in Pakistan and Native Pakistanis (Fig 2b). Within the latter cluster, the root and basal branches were occupied by Afghan refugees (PSP = 0.9) and all migration events were unidirectional (i.e., from Afghan refugees to Native Pakistanis). These observations suggested that the CRF35_AD_Afghan-Iranian_ strains were likely more closely related to A1 sequences circulating among Afghan refugees, rather than Native Pakistanis (Fig 2b).

The results of our sensitivity analyses suggested that the key parameters were robust to the choice of different datasets (S6 and S7 Figs). Moreover, similar to the complete analyses, we could not clearly identify the country (i.e., Afghanistan or Iran) that first established or received the CRF35_AD epidemic. In the complete analyses, Iran occupied the root and basal nodes of the CRF35_AD_Afghan-Iranian_ cluster. However, it is notable that some Afghan branches diverged directly from the root of this cluster (Fig 2a and 2c), which raise uncertainties regarding the true root location of the cluster. Similar pattern was observed in some of the balanced MCC trees as well (S7c Fig). In addition, in the majority of other balanced MCC trees, Afghanistan was the country that occupied the root and basal nodes of the CRF35_AD_Afghan-Iranian_ cluster (S7a, S7b and S7d Fig). This observation further highlighted the uncertainty surrounding the root location of the CRF35_AD_Afghan-Iranian_ cluster.

## Discussion

In this study, we applied a Bayesian phylogeographic approach to reconstruct the spatio-temporal history of the CRF35_AD clade for the first time. Our results suggested the epidemic in Afghanistan and Iran to have started at around 1990–1992 and showed an ongoing bidirectional dispersion of the virus between both countries. The CRF35_AD strains available from Afghan refugees living in Pakistan showed linkage to the Afghan-Iranian CRF35_AD epidemic, but the North American CRF35_AD-like sequence indicated independent evolutionary history. Among parental sequences available worldwide, only those from Kenya, Uganda and a cluster of Afghan refugees living in Pakistan showed significant genetic relationships with CRF35_AD sequences.

Mean evolutionary rates estimated in this study, were consistent with the rates estimated for the HIV-1 group M, under the *gag* and *pol* regions [29–38, 51–57]. The rate estimated for the *pol*_1 alignment was slightly higher than the one estimated for the *pol*_2 alignment. The *pol*_1 alignment included pure subtype D sequences while the *pol*_2 alignment included pure subtype A1 sequences; therefore, the difference in evolutionary rates between these alignments may be due to different evolutionary forces experienced by each parental clade [32, 58, 59].

Our analyses traced the *T*_*mrca*_ of the CRF35_AD_Iranian/Afghan_ cluster back to the early 1990s (1990–1992). Our analyses also set the lowest lower limit and highest upper limit of the 95% BCIs of the relevant dates for 1987 and 1994, respectively. In Afghanistan, the HIV epidemic was first identified in 1990, although the subtype of the identified cases remained unknown [60]. The first case with HIV-1 CRF35_AD infection was identified in this country in 2005 [10]. In Iran, HIV epidemic was first identified in 1987, among five hemophiliac patients. HIV-1 subtype B was later noticed to be the infecting lineage [5, 50]. In this country, HIV-1 CRF35_AD infection was first identified in 2002 [50]. The estimated onset date of the CRF35_AD_Afghan-Iranian_ epidemic is in line with the elevated opium and heroin production in Afghanistan, since the late 1970s [61], which suggests a potential association between the CRF35_AD epidemic and increased illicit drug use and trafficking. In our study, the estimated *T*_*mrca*_ of the Ugandan subtype A1 and D strains were consistent with previous estimates [62, 63], which also offers support for the validity of our models and dating estimates.

Two hypotheses have been proposed about the transmission order of the CRF35_AD between Afghanistan and Iran, both of which suggesting a unidirectional transmission of the virus across Afghanistan and Iran [8, 64]. Observing the existence of CRF35_AD in both countries, Mumtaz, et al. (2014) hypothesized that the epidemic in Afghanistan was driven by the return of Afghan refugees infected during their residence in Iran [64]. Conversely, Jahanbakhsh, et al. (2013) compared Afghan and Iranian CRF35_AD genome sequences and found two clusters containing both Afghan and Iranian strains. Observing such genetic similarities, and given the role of Afghanistan as the major heroin producer, they hypothesized that the virus may have migrated from Afghanistan to Iran through heroin trafficking routes [8]. Our results add a piece to this epidemiological puzzle, providing evidence that the spread of CRF35_AD in Afghanistan and Iran is not unidirectional and the virus may have circulated across these countries in a bidirectional manner.

Drug use and trafficking could be the major drivers of between-country virus exchange. In Iran and Afghanistan, CRF35_AD is mainly dominant among People Who Inject Drugs (PWIDs) [65, 66]. In our datasets, 72% of individuals infected with HIV-1 CRF35_AD, were PWIDs (S4 Table). For many years, Iran has been the primary trafficking route for heroin destined from Afghanistan to Europe, through the Balkan route [67]. However, since 2010, this pathway has gradually dried up due to Iran’s war on drugs policies [68]. Within drug trafficking routes, extended social networks of drug distributors in addition to their engagement in high-risk behaviours (e.g., injection drug use, needle exchange, unprotected sex, multiple partnerships, and frequent imprisonments), play a key role in the dynamics of HIV transmission across the borders [13]. This might explain the coincidence of the epidemic emergence and the opium production rise period. It may also help the virus to circulate among drug distributors, PWIDs, and their sexual partners across the countries.

Furthermore, mass migration of Afghan refugees to Iran since the rise of Taliban could have also contributed to between-country HIV transmission where Afghan refugees might have exchanged the virus with Iranians. Afghans make up the largest group of foreign nationals in Iran [69]. Our results suggest most viral exchange episodes to have occurred between 2000 and 2005, which coincides with the influx of Afghan refugees in Iran [70]. Moreover, some Afghans who showed evidence of between-country viral exchange in our MCC trees had previously migrated to Iran and Pakistan (accession numbers: GQ477446, GQ477448, and EF158040-42; S4 Table). Evidence for virus exchange between Afghan refugees and the host country (i.e., Pakistan) has been reported previously by Ansari, et al. (2011) [13]. Interestingly, our Bayesian phylogeographic analyses of the same strains used by Ansari, et al. suggested that the virus has probably transmitted unidirectionally from Afghan refugees to Pakistani individuals, through multiple transmission episodes (Fig 2b). This finding provides an example of “refugee-to-host” HIV transmission, which is in contrast to the previous speculations on the unidirectional transmission of HIV-1 from Iranians to Afghan refugees living in Iran [11, 64]. In a systematic review and meta-analysis study, Mostafavi, et al. (2015) found that a high proportion of Afghan migrants in Iran are infected with one or more major infectious diseases, such as multiple-drug-resistant tuberculosis (56%), malaria (40%), Crimean-Congo hemorrhagic fever (25%), and cholera (8%) [71]. Taken together, these observations suggest that screening newly arriving immigrants and refugees from Afghanistan for HIV could be beneficial.

The mass migration of illegal Afghan labours to Iran is another potential factor contributing to the HIV-1 exchange between Afghanistan and Iran. Iran is one of the main job markets for male Afghan labours, who frequently travel between the two countries [72]. Temporary migrant labours have been shown to engage in unprotected sex with female sex workers [73] and drug use practices [74, 75], presenting an opportunity for viral exchange between these subpopulations and reshaping the HIV epidemic in the host country. Establishment of screening programs for migrant Afghan labours may help to minimize onward transmissions. Improvement of healthcare measures and behavioural interventions targeting this group is also recommended.

Independent branching pattern and evolutionary history of the North American CRF35_AD-like sequence in the ML and the MCC tree suggest that this sequence may not be a true CRF35_AD lineage, but probably a URF_AD strain with its recombination breakpoints at the *pol* region being similar to the CRF35_AD. In the future, availability of full-length sequences of this type could shed more light on this preliminary hypothesis.

Our results also suggested that except A1 sequences from a community of Afghan refugees living in Pakistan, other neighboring countries with available parental A1 strains (i.e., Kazakhstan, Kuwait, Pakistan, Russia, Saudi Arabia, and Turkey) did not show evidence for genetic similarity with the CRF35_AD_Afghan-Iranian_ strains. This finding may not support the hypothesis that CRF35_AD has originated from these neighboring countries. However, this preliminary supposition should be further investigated when more sequences become available in the future.

In addition to the A1 sequences from Afghan refugees living in Pakistan, the CRF35_AD_Afghan-Iranian_ strains showed genetic similarity to parental A1 and D sequences circulating in Kenya and Uganda. However, given the lack of comprehensive samples from countries surrounding Afghanistan and Iran, it is not possible to identify if CRF35_AD sequences are directly stemmed from Kenya and Uganda or the transmission has occurred through intermediate countries. Moreover, given the lack of subtype D sequences from Afghan refugees living in Pakistan, it was not possible to conclude that CRF35_AD sequences were stemmed from this population. Overall, the abovementioned findings can only provide insight about the countries whose parental sequences are currently the closest strains to the CRF35_AD sequences, but investigation of the origin should be done once comprehensive samples become available from different countries of the region.

The results of the sensitivity analyses suggested that our key parameters (e.g., tree topology and height, CRF35_AD dating estimates, bidirectional transmission of the virus between Afghanistan and Iran, etc.) were robust to the choice of different datasets. Both in the complete and balanced analyses, we could not clearly identify the country (i.e, Afghanistan and Iran) that first established or received the CRF35_AD epidemic. Given that both Iranian and Afghan CRF35_AD sequences were of relatively similar genetic distance at the investigated genomic regions (Afghanistan: 0.023–0.045; Iran: 0.027–0.049), providing a robust estimate for this parameter was challenging. In the future, the use of more geographically balanced and geographically representative datasets, as well as longer or ideally full-length viral genomic regions, may help resolve this ambiguity.

Overall, although our findings provide novel contributions to the understanding of the dissemination of CRF35_AD clade across Afghanistan and Iran, they are subject to a number of limitations. Given the limited available geo-referenced sequence data, especially from the neighboring countries, we could not investigate the CRF35_AD clade’s place and date of birth and clarify the source of the epidemic in Afghanistan and Iran. In addition, we could not estimate the evolutionary rates directly from the CRF35_AD alignments as the time-span of these datasets were not wide enough. In future, our model and dating estimates should be further investigated as new sequences from additional countries and time-spans become available. On the other hand, our phylogeographic analyses were based on multiple loci covering both parental subtypes of the CRF35_AD clade (i.e., A1 and D). This allowed for a comprehensive evaluation of the CRF35_AD clade’s evolutionary history.

## Conclusion

In summary, our results suggested that the CRF35_AD epidemic in Afghanistan and Iran may have started at around 1990 and 1992 and disseminated between both countries in a bidirectional manner. The CRF35_AD epidemic among Afghan refugees living in Pakistan also showed epidemiological links to the Afghan-Iranian CRF35_AD epidemic. However, the North American CRF35_AD-like strain showed an independent evolutionary history, suggesting that it may not be a true CRF35_AD lineage. Potential factors contributing to viral exchange between Afghanistan and Iran seem to be injection drug networks and mass migration of Afghan refugees and labours to Iran. Screening of newly arriving immigrants and refugees for HIV may help minimize onward transmissions. Improvement of healthcare measures and behavioural interventions targeting these groups is also recommended. Upon availability of further samples, additional studies will be necessary to identify the origin of the CRF35_AD clade and the source of the epidemic in Afghanistan and Iran.

## Supporting Information

S1 Fig ML phylogenetic analysis of CRF35_AD and parental subtypes A1/D. (PDF)

S2 Fig Identification of genetically similar parental strains. (PDF)

S3 Fig Substitution saturation analysis. (PDF)

S4 Fig Likelihood mapping analysis. (PDF)

S5 Fig Genetic characterization of CRF35_AD sequences. (PDF)

S6 Fig Robustness of the CRF35_AD_Afghan-Iranian_ cluster’s dating estimates to the choice of different datasets. (PDF)

S7 Fig Robustness of the key parameters’ estimates to the choice of different datasets. (PDF)

S1 Table HIV-1 CRF35_AD datasets used in this study. (PDF)

S2 Table HIV-1 parental A1 and D sequences used in Bayesian phylogeographic analyses. (PDF)

S3 Table Complete and country-balanced CRF35_AD datasets used for sensitivity analyses. (PDF)

S4 Table Accession numbers of HIV-1 sequences used in this study. (XLSX)
