# Supplementary material for: Spatio-Temporal History of HIV-1 CRF35_AD in Afghanistan and Iran
Source: PLoS One. 2016 Jun 9;11(6):e0156499. doi: 10.1371/journal.pone.0156499 (PMC4900578; doi:10.1371/journal.pone.0156499)
Supplement: S1 Fig — (PDF) [file pone.0156499.s001.pdf]

a) *gag*_1

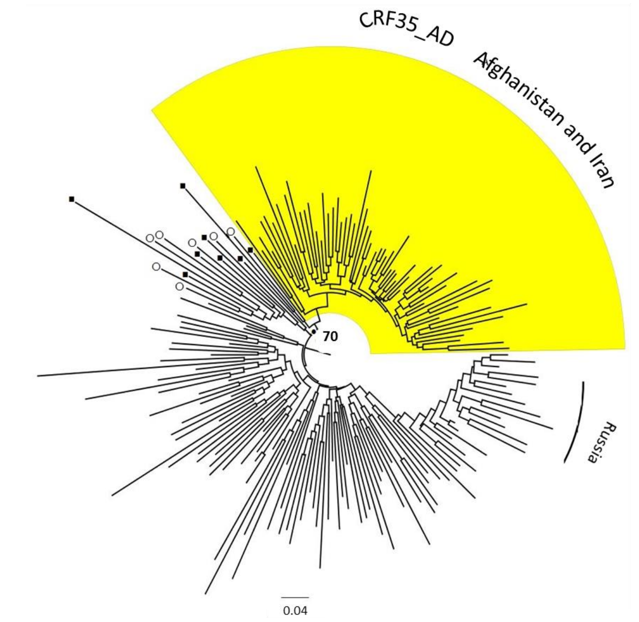

b) *gag*_2

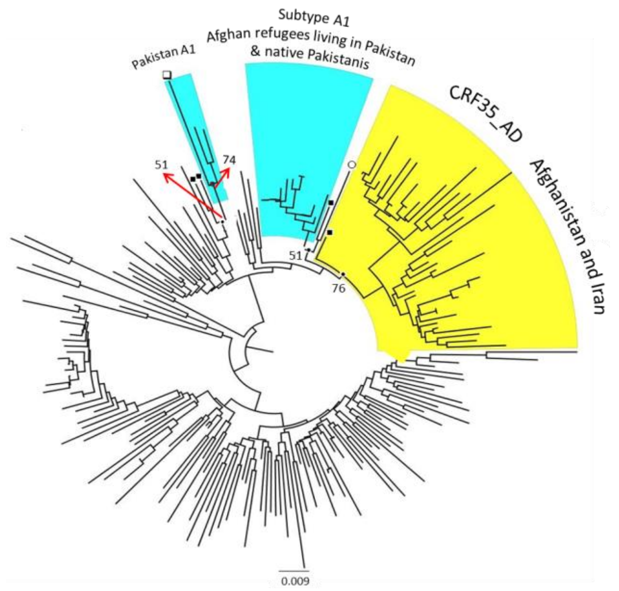

c) *pol*_1

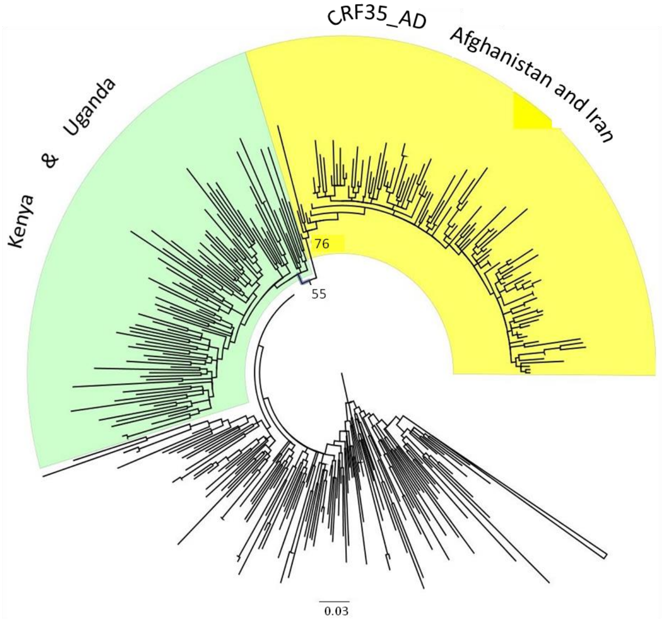

d) *pol*_2

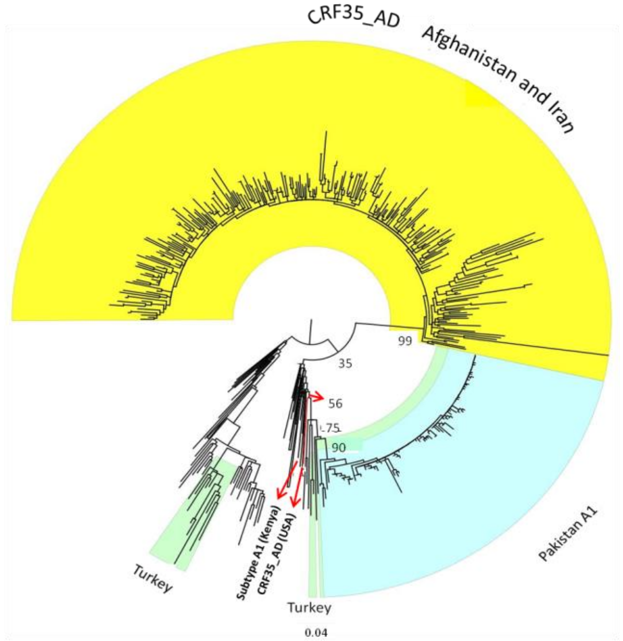

S1 Fig. ML phylogenetic analysis of CRF35_AD and parental subtypes A1/D. (a) *gag*_1 region: countries with available subtype A1 sequences in this genomic region included Cyprus, Kazakhstan, Kenya, Russia, Rwanda, Senegal, Sweden, Tanzania, Uganda, and Ukraine. CRF35_AD sequences at this genomic region were available from Afghanistan and Iran; **(b) *gag*_2 region:** countries with available subtype A1 sequences in this genomic region included Cyprus, Kazakhstan, Kenya, Pakistan, Russia, Rwanda, Saudi Arabia, Senegal, Sweden, Tanzania, Uganda, and Ukraine. CRF35_AD sequences at this genomic region were available from Afghanistan, Iran, and a sample of Afghan refugees living in Pakistan; **(c) *pol*_1 region:** countries with available subtype D sequences in this genomic region included Cameroon, Democratic Republic of Congo, France, Kenya, Senegal, South Africa, Sudan, Tanzania, and Uganda.

CRF35_AD sequences at this genomic region were available from Afghanistan and Iran; **(d)** ***pol*****_2 region:** countries with available subtype A1 sequences under this genomic region included Kazakhstan, Kenya, Kuwait, Pakistan, Russia, Senegal, Thailand, Turkey, and Uganda. CRF35_AD sequences at this genomic region were available from Afghanistan, Iran, and USA. Across all phylogenies, CRF35_AD sequences (except the one from USA) made a monophyletic cluster (highlighted in yellow). CRF35_AD clusters demonstrated genetic similarity to parental sequences from Kenya and Uganda (Part a, b, and c) and a cluster of Afghan refugees and Native Pakistanis (Part b). In *pol*_2 phylogeny (Part d), none of the parental sequences showed genetic similarity to $\text{CRF35\_AD}_{\text{Afghan-Iranian}}$ cluster. But, the CRF35_AD-like sequence available from USA joined independently with a Kenyan subtype A1 sequence (Bootstrap= 56%, Part d). Despite a set of A1 sequences available from Afghan refugees and Native Pakistanis, parental sequences from other neighboring countries did not demonstrate genetic similarity to CRF35_AD sequences. These countries included Kazakhstan, Kuwait, Russia, Saudi Arabia, and Turkey. □ Saudi Arabia ○ Uganda ▪ Kenya
