# Supplementary material for: Spatio-Temporal History of HIV-1 CRF35_AD in Afghanistan and Iran
Source: PLoS One. 2016 Jun 9;11(6):e0156499. doi: 10.1371/journal.pone.0156499 (PMC4900578; doi:10.1371/journal.pone.0156499)
Supplement: S2 Fig — (PDF) [file pone.0156499.s002.pdf]

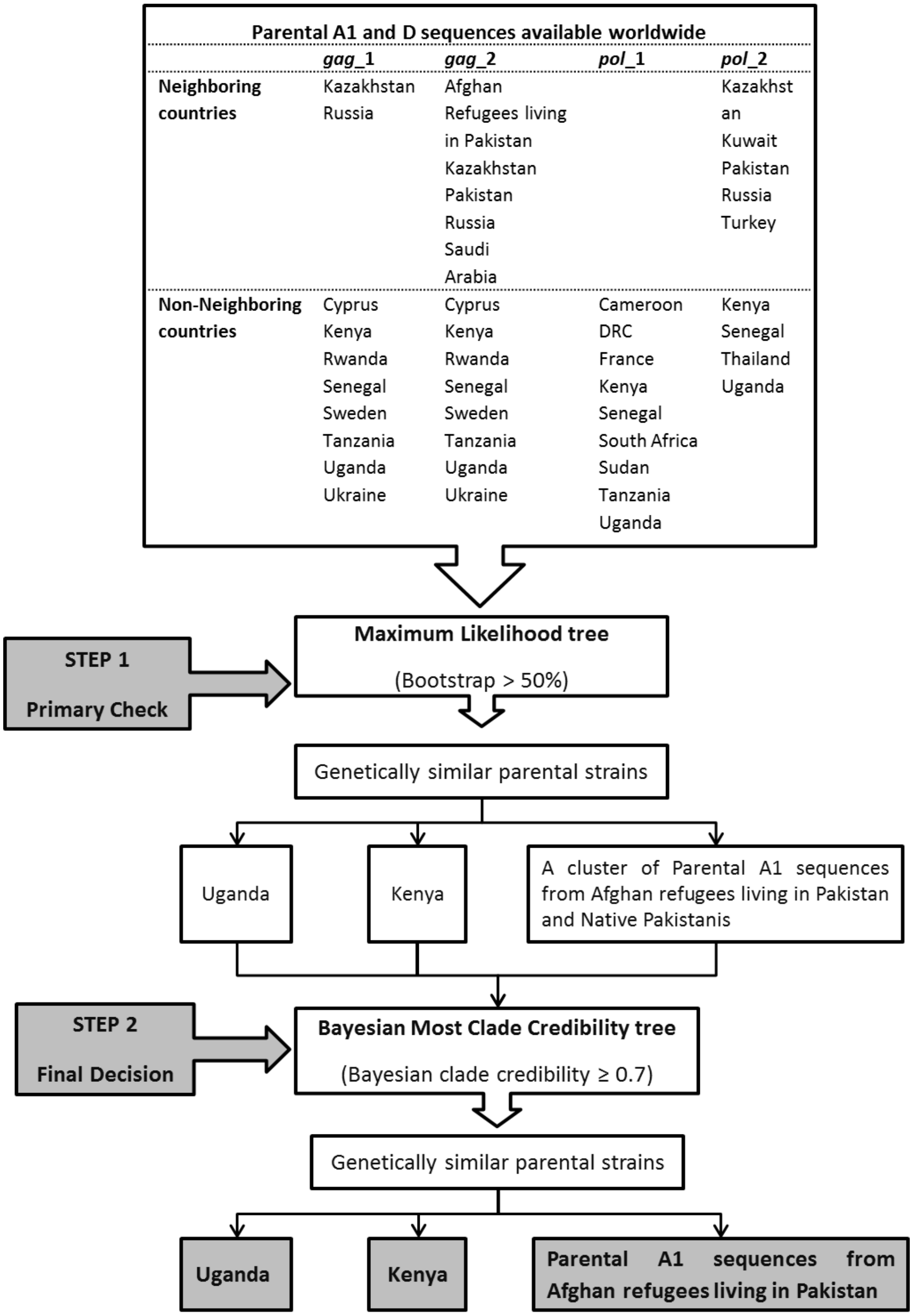

S2 Fig. Identification of genetically similar parental strains. Under each genomic region, all parental strains available worldwide were tested with maximum likelihood phylogenetic tree (STEP 1). Those strains demonstrating minimum evidence of genetic similarity to CRF35_AD clusters (bootstrap > 50%) at this step were reassessed by Bayesian phylogenetic trees (STEP 2). At this step, a higher threshold value was considered for assessing clade support (Posterior Clade Credibility > 0.7). Using these criteria, CRF35_AD strains showed genetic similarity to parental strains from Uganda, Kenya, and a community of Afghan refugees living in Pakistan. **DRC:** Democratic Republic of Congo
