# Supplementary material for: Spatio-Temporal History of HIV-1 CRF35_AD in Afghanistan and Iran
Source: PLoS One. 2016 Jun 9;11(6):e0156499. doi: 10.1371/journal.pone.0156499 (PMC4900578; doi:10.1371/journal.pone.0156499)
Supplement: S3 Fig — (PDF) [file pone.0156499.s003.pdf]

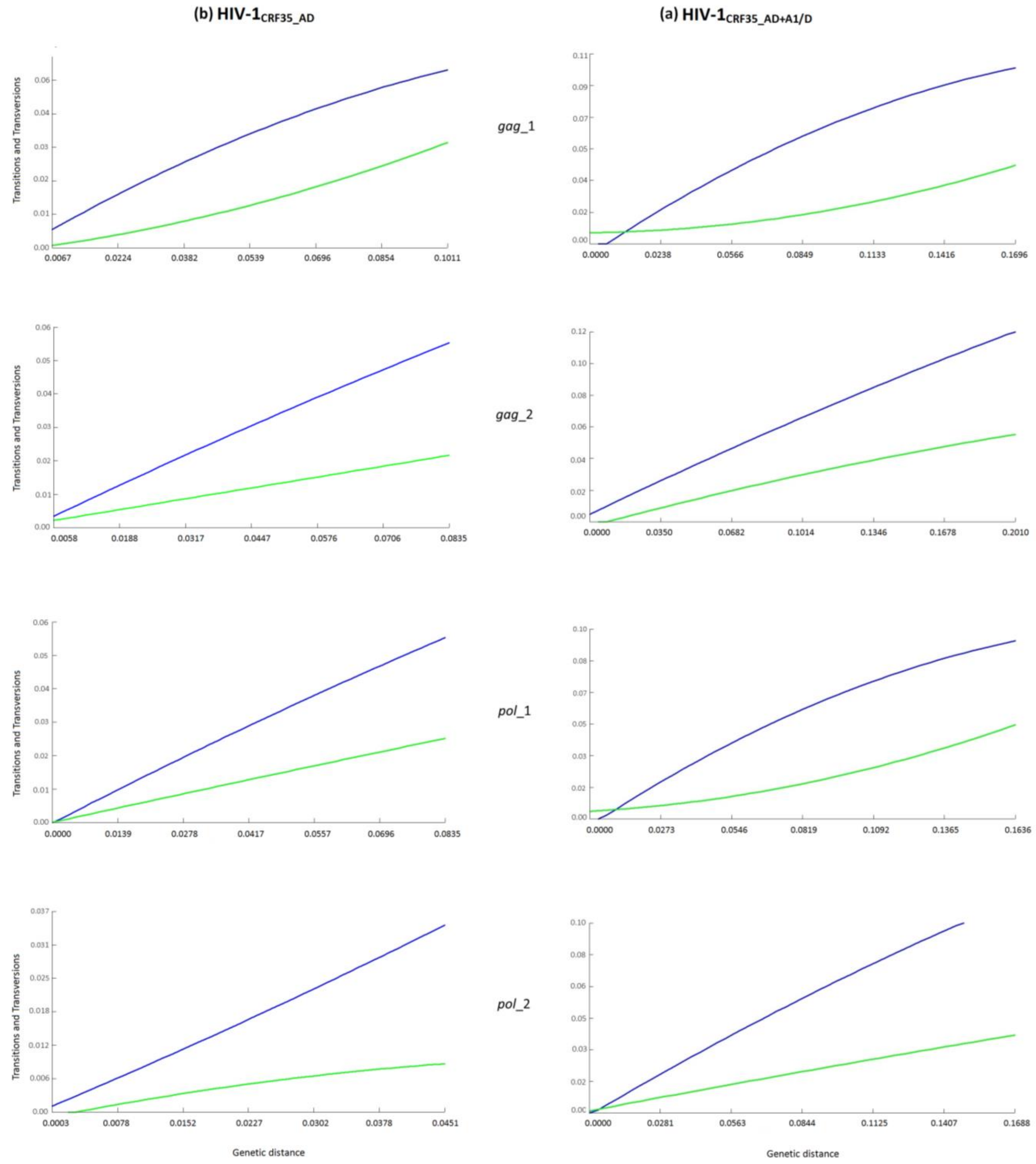

S3 Fig. Substitution saturation analysis. (a) HIV-1_{CRF35_AD} datasets, **(b)** HIV-$1_{CRF35\_AD+A1/D}$ datasets. Transitions (blue line) and Transversions (green line) are plotted against divergence. The genetic distance is computed with the GTR nucleotide substitution model. The results show that all the datasets contain enough evolutionary information for reliable phylogenetic and molecular clock inferences.
