# Supplementary material for: Spatio-Temporal History of HIV-1 CRF35_AD in Afghanistan and Iran
Source: PLoS One. 2016 Jun 9;11(6):e0156499. doi: 10.1371/journal.pone.0156499 (PMC4900578; doi:10.1371/journal.pone.0156499)
Supplement: S4 Fig — (PDF) [file pone.0156499.s004.pdf]

b) HIV-1$_{CRF35_AD}$

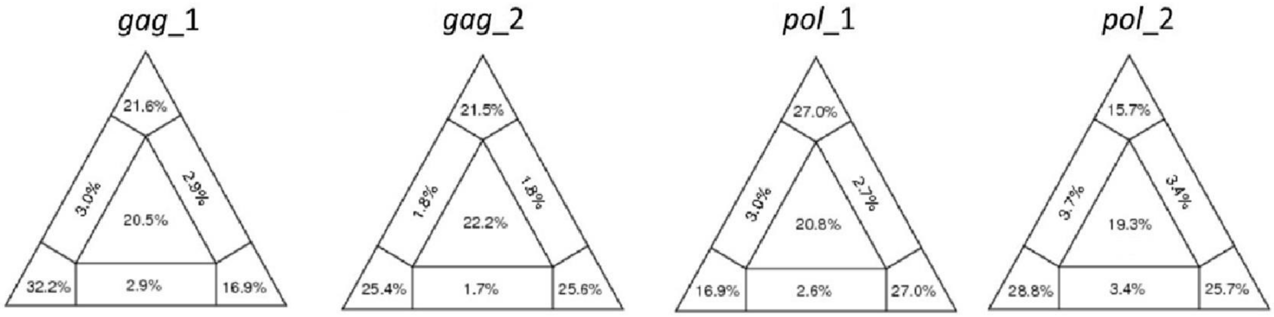

a) HIV-1$_{CRF35_AD+A1/D}$

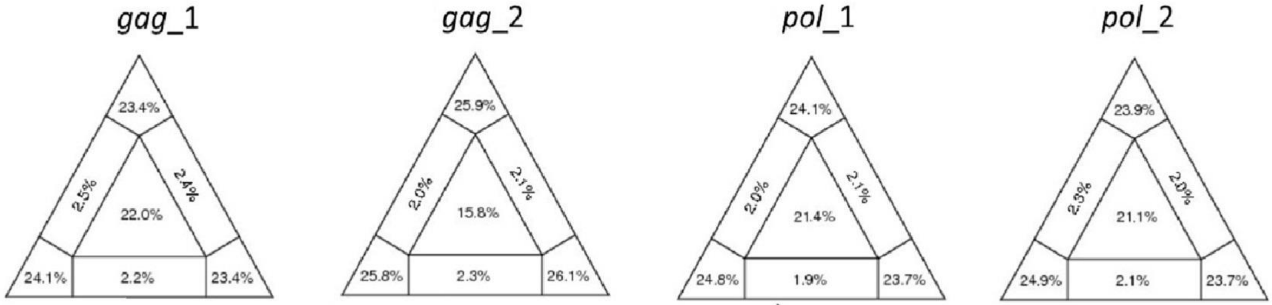

S4 Fig. Likelihood mapping analysis. (a) HIV-1$_{CRF35_AD}$ datasets, **(b)** HIV-1$_{CRF35_AD+A1/D}$ datasets**.** The analyses showed that, across all datasets, less than 30% of trees [1] are unresolved or semi-resolved. This suggests the sufficiency of phylogenetic signal in our datasets. Analyses were performed using 10,000 random quartets and a Gamma model of rate heterogeneity (G8).

Reference

1. Lemey P, Salemi M, and Vandamme AM, *The Phylogenetic Handbook. A Practical Approach to Phylogenetic Analysis and Hypothesis Testing*. second ed. Phylogenetic inference using maximum likelihood methods, ed. Schmidt HA and Haeseler A2009, london: Cambridge University Press.
