# Supplementary material for: Spatio-Temporal History of HIV-1 CRF35_AD in Afghanistan and Iran
Source: PLoS One. 2016 Jun 9;11(6):e0156499. doi: 10.1371/journal.pone.0156499 (PMC4900578; doi:10.1371/journal.pone.0156499)
Supplement: S5 Fig — (PDF) [file pone.0156499.s005.pdf]

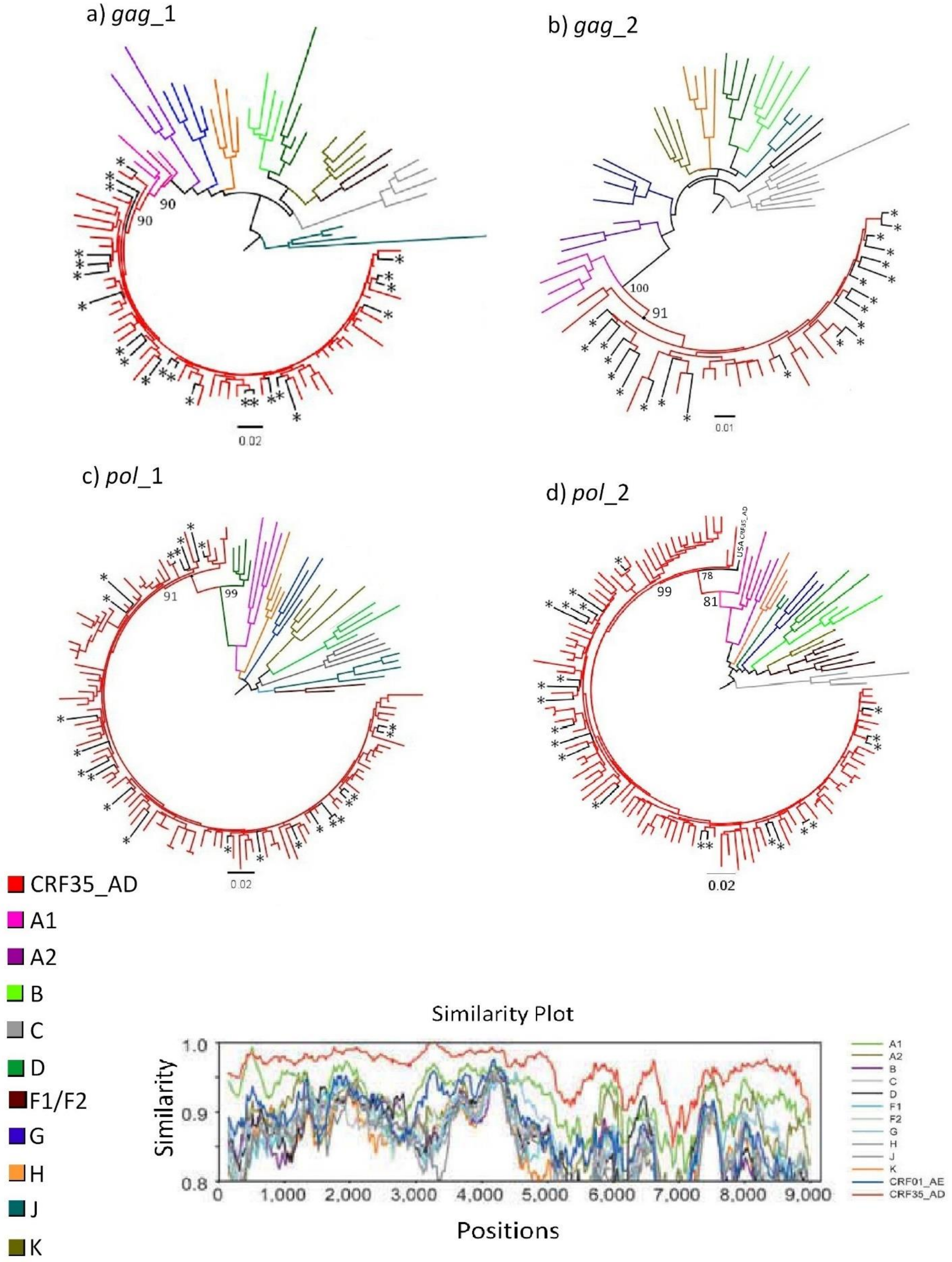

a) gag_1
b) gag_2
c) pol_1
d) pol_2
90
90
100
91
91
99
99
78
81
USA CRF35_AD
0.02
0.01
0.02
0.02
CRF35_AD
A1
A2
B
C
D
F1/F2
G
H
J
K
Similarity Plot
Similarity
Positions
1.0
0.9
0.8
0
1,000
2,000
3,000
4,000
5,000
6,000
7,000
8,000
9,000
A1
A2
B
C
D
F1
F2
G
H
J
K
CRF01_AE
CRF35_AD

S5 Fig. Genetic characterization of CRF35_AD sequences. Phylogenetic trees represent ML tree of the HIV-1 CRF35_AD *gag* **(a-b)** and *pol* **(c-d)** sequences and pure subtype reference sequences selected from the Los Alamos HIV database. The trees represent clustering of the sequences with subtype A1 reference sequences in (a-b and d) and with subtype D reference sequences in (c). CRF35_AD reference sequences (EF158040-43) as well as full-length sequences that we have characterized by similarity plotting, are interspersed among CRF35_AD-like sequences and are marked with asterisks. The color code is indicated in the legend, on the lower left corner. The bootstrap values (1000 replicates) are indicated as percents. The trees are midpoint-rooted and scaled. A representative similarity plot of a full-length CRF35_AD sequence is depicted in the bottom of the figure (Accession Number: AB703607).
