# Supplementary material for: Spatio-Temporal History of HIV-1 CRF35_AD in Afghanistan and Iran
Source: PLoS One. 2016 Jun 9;11(6):e0156499. doi: 10.1371/journal.pone.0156499 (PMC4900578; doi:10.1371/journal.pone.0156499)
Supplement: S6 Fig — (PDF) [file pone.0156499.s006.pdf]

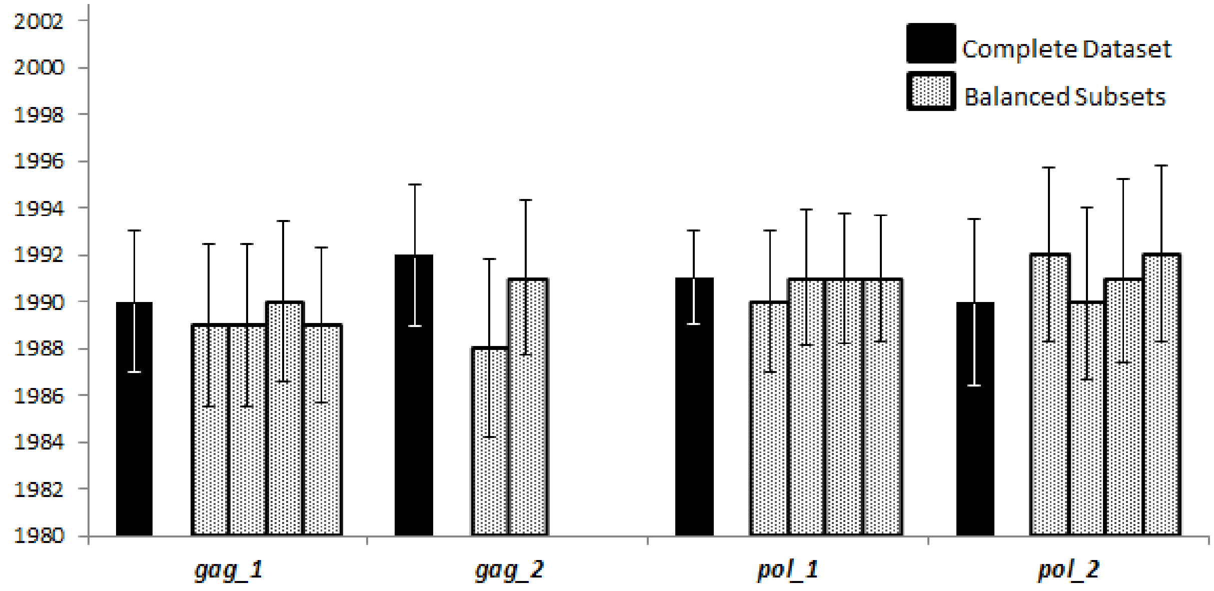

S6 Fig. Robustness of the CRF35_AD$_{\text{Afghan-Iranian}}$ cluster's dating estimates to the choice of different datasets. Vertical box refers to mean parameter estimates and error bars represent the corresponding 95% Bayesian credible intervals. As shown in the figure, Bayesian credible intervals of all dating estimates overlap, supporting the robustness of this parameter to the choice of different datasets. In the *gag*_2 region, the total number of sequences available from Iran was small (n=21); therefore, only two balanced subsets were created and analyzed for this region. The color code in the upper right corner indicates the type of datasets.
