# Supplementary material for: Spatio-Temporal History of HIV-1 CRF35_AD in Afghanistan and Iran
Source: PLoS One. 2016 Jun 9;11(6):e0156499. doi: 10.1371/journal.pone.0156499 (PMC4900578; doi:10.1371/journal.pone.0156499)
Supplement: S7 Fig — (PDF) [file pone.0156499.s007.pdf]

a) ***gag*_1**

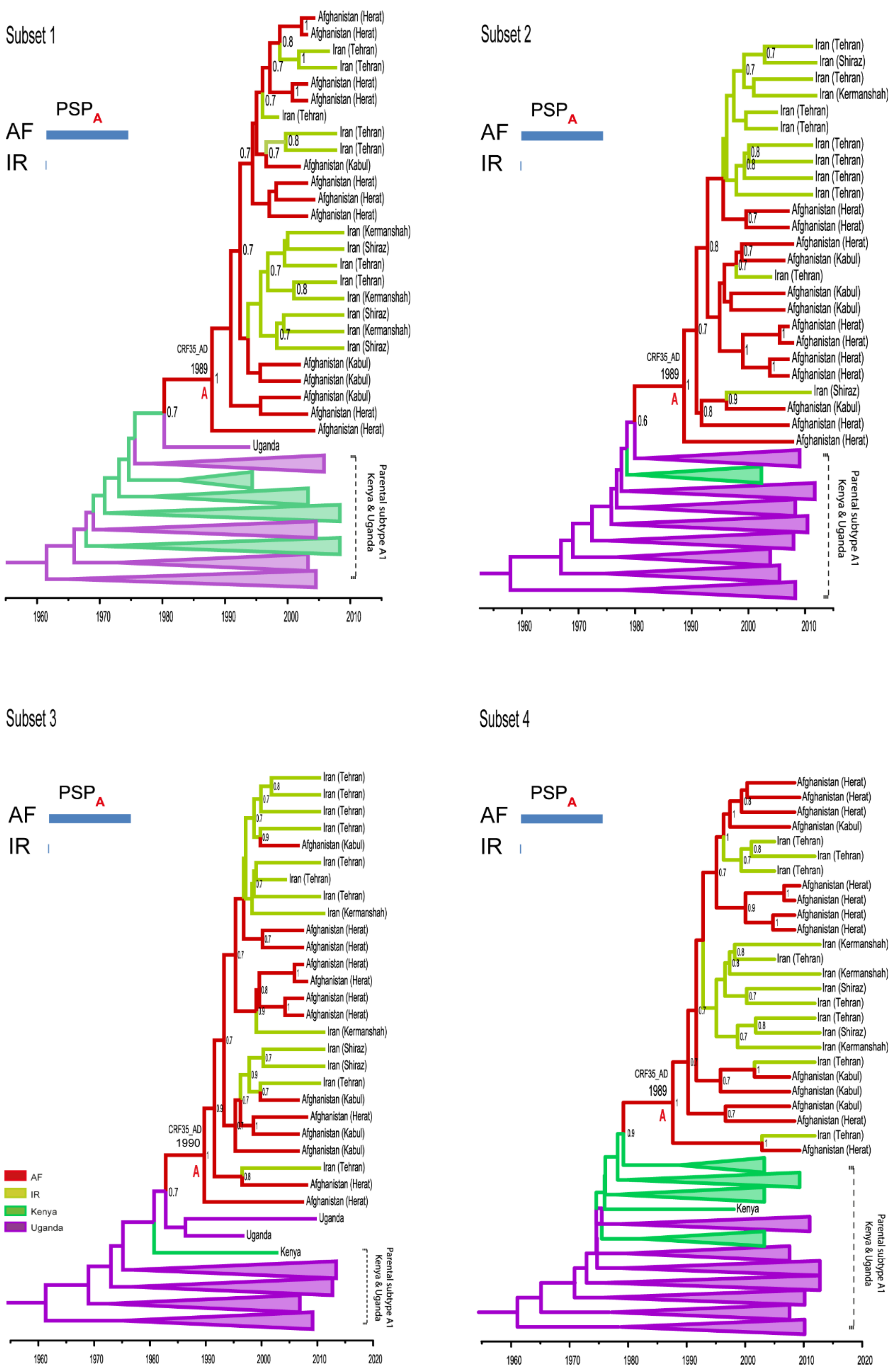

Subset 1
PSPA
AF
IR
Afghanistan (Herat)
Afghanistan (Herat)
Iran (Tehran)
Iran (Tehran)
Afghanistan (Herat)
Afghanistan (Herat)
Iran (Tehran)
Iran (Tehran)
Iran (Tehran)
Afghanistan (Kabul)
Afghanistan (Herat)
Afghanistan (Herat)
Afghanistan (Herat)
Iran (Kermanshah)
Iran (Shiraz)
Iran (Tehran)
Iran (Tehran)
Iran (Kermanshah)
Iran (Shiraz)
Iran (Kermanshah)
Iran (Shiraz)
Afghanistan (Kabul)
Afghanistan (Kabul)
Afghanistan (Kabul)
Afghanistan (Herat)
Afghanistan (Herat)
CRF35_AD
1989
A
Uganda
Parental subtype A1
Kenya & Uganda
1960 1970 1980 1990 2000 2010
Subset 2
PSPA
AF
IR
Iran (Tehran)
Iran (Shiraz)
Iran (Tehran)
Iran (Kermanshah)
Iran (Tehran)
Iran (Tehran)
Iran (Tehran)
Iran (Tehran)
Iran (Tehran)
Iran (Tehran)
Afghanistan (Herat)
Afghanistan (Herat)
Afghanistan (Herat)
Afghanistan (Kabul)
Iran (Tehran)
Afghanistan (Kabul)
Afghanistan (Kabul)
Afghanistan (Herat)
Afghanistan (Herat)
Afghanistan (Herat)
Afghanistan (Herat)
Iran (Shiraz)
Afghanistan (Kabul)
Afghanistan (Herat)
Afghanistan (Herat)
CRF35_AD
1989
A
Parental subtype A1
Kenya & Uganda
1960 1970 1980 1990 2000 2010
Subset 3
PSPA
AF
IR
Iran (Tehran)
Iran (Tehran)
Iran (Tehran)
Iran (Tehran)
Afghanistan (Kabul)
Iran (Tehran)
Iran (Tehran)
Iran (Tehran)
Iran (Kermanshah)
Afghanistan (Herat)
Afghanistan (Herat)
Afghanistan (Herat)
Afghanistan (Herat)
Afghanistan (Herat)
Afghanistan (Herat)
Iran (Kermanshah)
Iran (Shiraz)
Iran (Shiraz)
Iran (Tehran)
Afghanistan (Kabul)
Afghanistan (Herat)
Afghanistan (Kabul)
Afghanistan (Kabul)
Iran (Tehran)
Afghanistan (Herat)
Afghanistan (Herat)
CRF35_AD
1990
A
Uganda
Uganda
Kenya
AF
IR
Kenya
Uganda
Parental subtype A1
Kenya & Uganda
1960 1970 1980 1990 2000 2010 2020
Subset 4
PSPA
AF
IR
Afghanistan (Herat)
Afghanistan (Herat)
Afghanistan (Herat)
Afghanistan (Kabul)
Iran (Tehran)
Iran (Tehran)
Iran (Tehran)
Afghanistan (Herat)
Afghanistan (Herat)
Afghanistan (Herat)
Afghanistan (Herat)
Iran (Kermanshah)
Iran (Tehran)
Iran (Kermanshah)
Iran (Shiraz)
Iran (Tehran)
Iran (Tehran)
Iran (Shiraz)
Iran (Kermanshah)
Iran (Tehran)
Afghanistan (Kabul)
Afghanistan (Kabul)
Afghanistan (Kabul)
Afghanistan (Herat)
Iran (Tehran)
Afghanistan (Herat)
CRF35_AD
1989
A
Kenya
Parental subtype A1
Kenya & Uganda
1960 1970 1980 1990 2000 2010 2020

b) *gag_2*

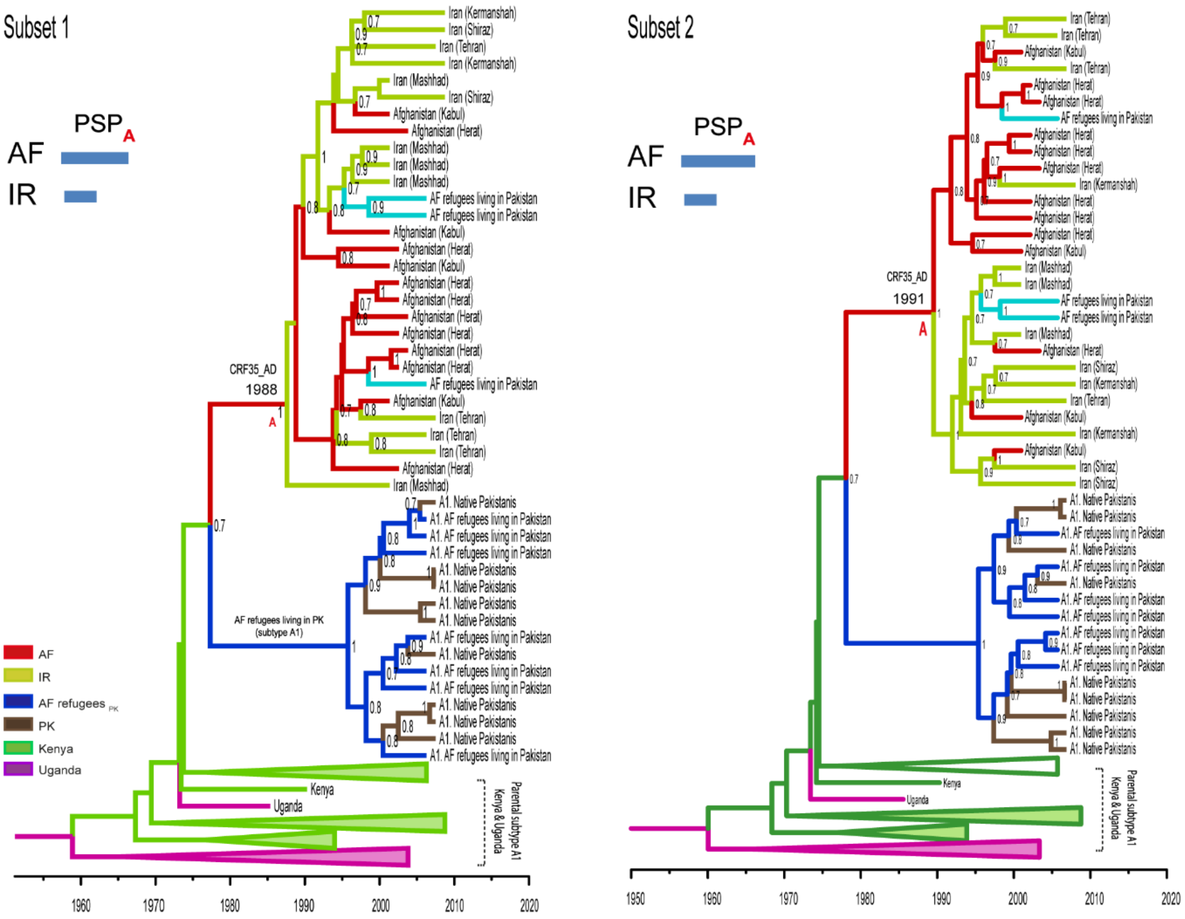

Subset 1
Subset 2
PSP A
AF
IR
CRF35_AD
1988
1991
AF refugees living in PK (subtype A1)
AF
IR
AF refugees PK
PK
Kenya
Uganda
Parental subtype A1 Kenya & Uganda

c) *pol*_1

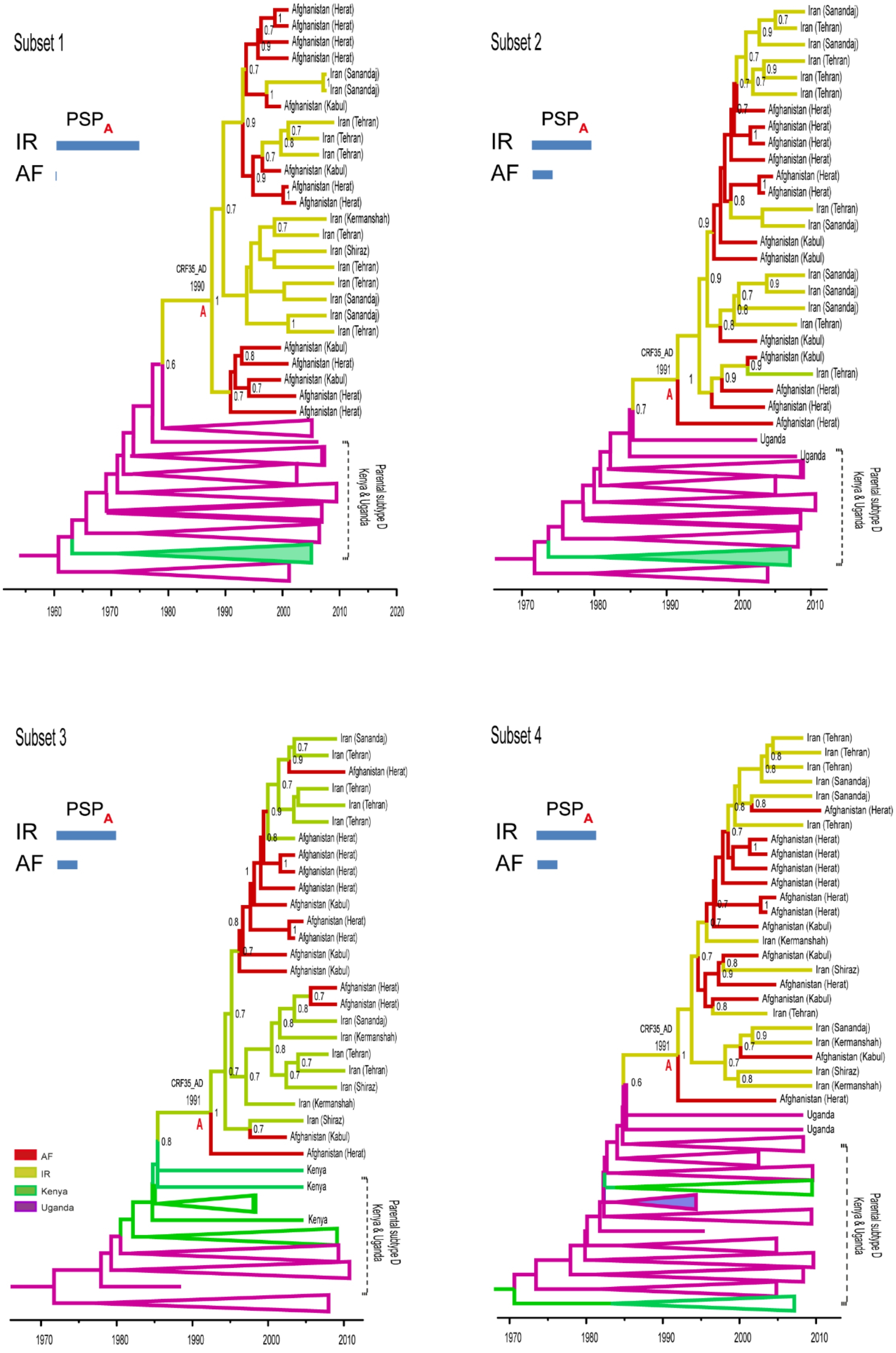
Subset 1
PSP A
IR
AF
Afghanistan (Herat)
Afghanistan (Herat)
Afghanistan (Herat)
Afghanistan (Herat)
Iran (Sanandaj)
Iran (Sanandaj)
Afghanistan (Kabul)
Iran (Tehran)
Iran (Tehran)
Iran (Tehran)
Afghanistan (Kabul)
Afghanistan (Herat)
Afghanistan (Herat)
Iran (Kermanshah)
Iran (Tehran)
Iran (Shiraz)
Iran (Tehran)
Iran (Tehran)
Iran (Sanandaj)
Iran (Sanandaj)
Iran (Tehran)
Afghanistan (Kabul)
Afghanistan (Herat)
Afghanistan (Kabul)
Afghanistan (Herat)
Afghanistan (Herat)
CRF35_AD
1990
A
Parental subtype D
Kenya & Uganda
1960
1970
1980
1990
2000
2010
2020
Subset 2
PSP A
IR
AF
Iran (Sanandaj)
Iran (Tehran)
Iran (Sanandaj)
Iran (Tehran)
Iran (Tehran)
Iran (Tehran)
Afghanistan (Herat)
Afghanistan (Herat)
Afghanistan (Herat)
Afghanistan (Herat)
Afghanistan (Herat)
Afghanistan (Herat)
Iran (Tehran)
Iran (Sanandaj)
Afghanistan (Kabul)
Afghanistan (Kabul)
Iran (Sanandaj)
Iran (Sanandaj)
Iran (Sanandaj)
Iran (Tehran)
Afghanistan (Kabul)
Afghanistan (Kabul)
Iran (Tehran)
Afghanistan (Herat)
Afghanistan (Herat)
Afghanistan (Herat)
Uganda
Uganda
CRF35_AD
1991
A
Parental subtype D
Kenya & Uganda
1970
1980
1990
2000
2010
Subset 3
PSP A
IR
AF
Iran (Sanandaj)
Iran (Tehran)
Afghanistan (Herat)
Iran (Tehran)
Iran (Tehran)
Iran (Tehran)
Afghanistan (Herat)
Afghanistan (Herat)
Afghanistan (Herat)
Afghanistan (Herat)
Afghanistan (Kabul)
Afghanistan (Herat)
Afghanistan (Herat)
Afghanistan (Kabul)
Afghanistan (Kabul)
Afghanistan (Herat)
Afghanistan (Herat)
Iran (Sanandaj)
Iran (Kermanshah)
Iran (Tehran)
Iran (Tehran)
Iran (Shiraz)
Iran (Kermanshah)
Iran (Shiraz)
Afghanistan (Kabul)
Afghanistan (Herat)
Kenya
Kenya
Kenya
CRF35_AD
1991
A
AF
IR
Kenya
Uganda
Parental subtype D
Kenya & Uganda
1970
1980
1990
2000
2010
Subset 4
PSP A
IR
AF
Iran (Tehran)
Iran (Tehran)
Iran (Tehran)
Iran (Sanandaj)
Iran (Sanandaj)
Afghanistan (Herat)
Iran (Tehran)
Afghanistan (Herat)
Afghanistan (Herat)
Afghanistan (Herat)
Afghanistan (Herat)
Afghanistan (Herat)
Afghanistan (Herat)
Afghanistan (Kabul)
Iran (Kermanshah)
Afghanistan (Kabul)
Iran (Shiraz)
Afghanistan (Herat)
Afghanistan (Kabul)
Iran (Tehran)
Iran (Sanandaj)
Iran (Kermanshah)
Afghanistan (Kabul)
Iran (Shiraz)
Iran (Kermanshah)
Afghanistan (Herat)
Uganda
Uganda
CRF35_AD
1991
A
Parental subtype D
Kenya & Uganda
1970
1980
1990
2000
2010

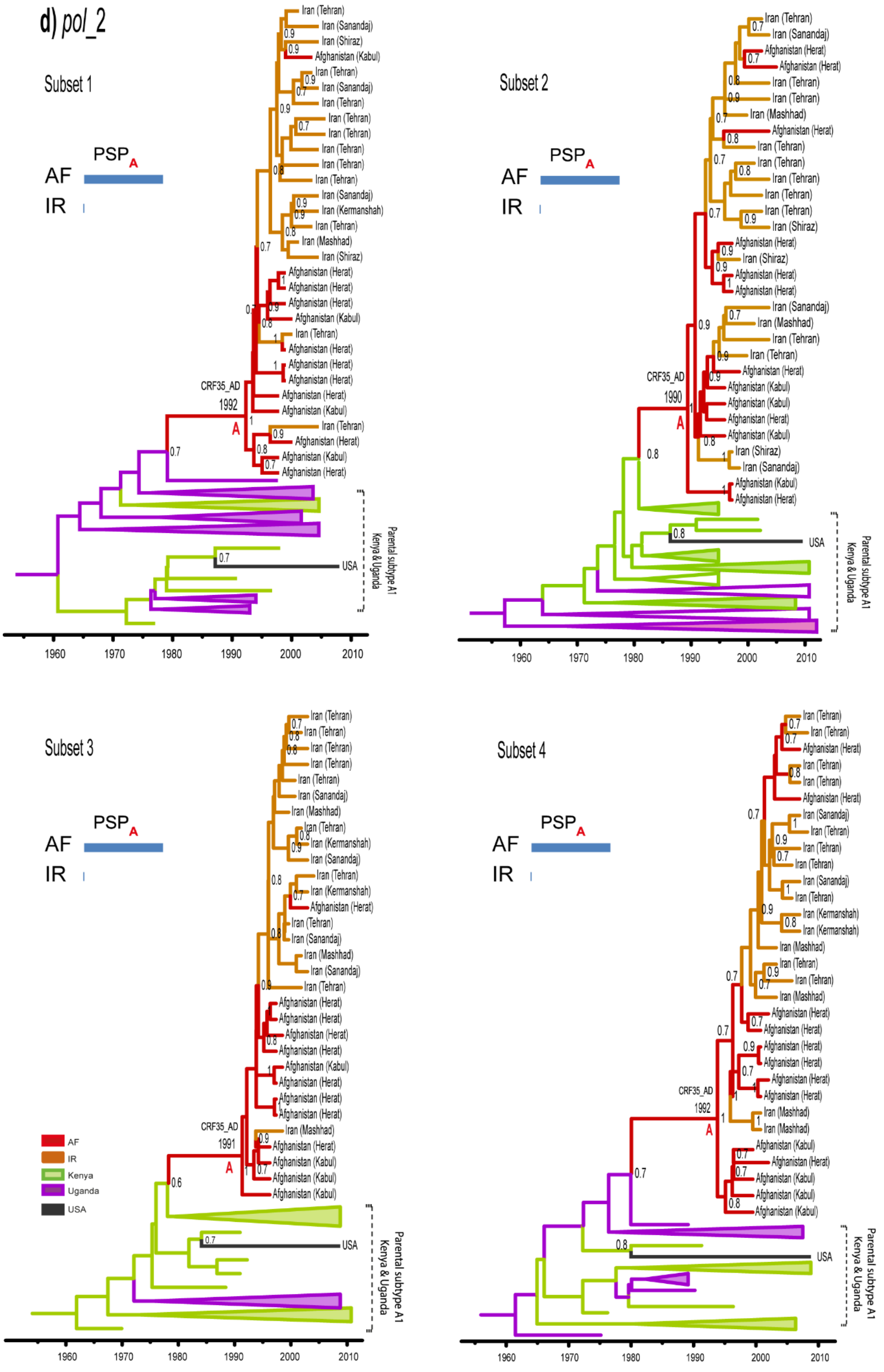

d) pol_2
Subset 1
PSP A
AF
IR
Iran (Tehran)
Iran (Sanandaj)
Iran (Shiraz)
Afghanistan (Kabul)
Iran (Tehran)
Iran (Sanandaj)
Iran (Tehran)
Iran (Tehran)
Iran (Tehran)
Iran (Tehran)
Iran (Tehran)
Iran (Tehran)
Iran (Sanandaj)
Iran (Kermanshah)
Iran (Tehran)
Iran (Mashhad)
Iran (Shiraz)
Afghanistan (Herat)
Afghanistan (Herat)
Afghanistan (Herat)
Afghanistan (Kabul)
Iran (Tehran)
Afghanistan (Herat)
Afghanistan (Herat)
Afghanistan (Herat)
Afghanistan (Herat)
Afghanistan (Kabul)
Iran (Tehran)
Afghanistan (Herat)
Afghanistan (Kabul)
Afghanistan (Herat)
CRF35_AD
1992
A
USA
Parental subtype A1
Kenya & Uganda
1960
1970
1980
1990
2000
2010
Subset 2
PSP A
AF
IR
Iran (Tehran)
Iran (Sanandaj)
Afghanistan (Herat)
Afghanistan (Herat)
Iran (Tehran)
Iran (Tehran)
Iran (Mashhad)
Afghanistan (Herat)
Iran (Tehran)
Iran (Tehran)
Iran (Tehran)
Iran (Tehran)
Iran (Tehran)
Iran (Shiraz)
Afghanistan (Herat)
Iran (Shiraz)
Afghanistan (Herat)
Afghanistan (Herat)
Iran (Sanandaj)
Iran (Mashhad)
Iran (Tehran)
Iran (Tehran)
Afghanistan (Herat)
Afghanistan (Kabul)
Afghanistan (Kabul)
Afghanistan (Herat)
Afghanistan (Kabul)
Iran (Shiraz)
Iran (Sanandaj)
Afghanistan (Kabul)
Afghanistan (Herat)
CRF35_AD
1990
A
USA
Parental subtype A1
Kenya & Uganda
1960
1970
1980
1990
2000
2010
Subset 3
PSP A
AF
IR
Iran (Tehran)
Iran (Tehran)
Iran (Tehran)
Iran (Tehran)
Iran (Tehran)
Iran (Sanandaj)
Iran (Mashhad)
Iran (Tehran)
Iran (Kermanshah)
Iran (Sanandaj)
Iran (Tehran)
Iran (Kermanshah)
Afghanistan (Herat)
Iran (Tehran)
Iran (Sanandaj)
Iran (Mashhad)
Iran (Sanandaj)
Iran (Tehran)
Afghanistan (Herat)
Afghanistan (Herat)
Afghanistan (Herat)
Afghanistan (Herat)
Afghanistan (Kabul)
Afghanistan (Herat)
Afghanistan (Herat)
Afghanistan (Herat)
Iran (Mashhad)
Afghanistan (Herat)
Afghanistan (Kabul)
Afghanistan (Kabul)
Afghanistan (Kabul)
CRF35_AD
1991
A
AF
IR
Kenya
Uganda
USA
Parental subtype A1
Kenya & Uganda
1960
1970
1980
1990
2000
2010
Subset 4
PSP A
AF
IR
Iran (Tehran)
Iran (Tehran)
Afghanistan (Herat)
Iran (Tehran)
Iran (Tehran)
Afghanistan (Herat)
Iran (Sanandaj)
Iran (Tehran)
Iran (Tehran)
Iran (Tehran)
Iran (Sanandaj)
Iran (Tehran)
Iran (Kermanshah)
Iran (Kermanshah)
Iran (Mashhad)
Iran (Tehran)
Iran (Tehran)
Iran (Mashhad)
Afghanistan (Herat)
Afghanistan (Herat)
Afghanistan (Herat)
Afghanistan (Herat)
Afghanistan (Herat)
Afghanistan (Herat)
Iran (Mashhad)
Iran (Mashhad)
Afghanistan (Kabul)
Afghanistan (Herat)
Afghanistan (Kabul)
Afghanistan (Kabul)
Afghanistan (Kabul)
CRF35_AD
1992
A
USA
Parental subtype A1
Kenya & Uganda
1960
1970
1980
1990
2000
2010

S7 Fig. Robustness of the key parameters' estimates to the choice of different datasets. (a) *gag*_1; (b) *gag*_2; (c) *pol*_1; (d) *pol_2*. As shown in the figure, the key parameters are robust to the choice of different datasets, including the tree topology and height, the T_{mrca} of the CRF35_AD$_{\text{Afghan-Iranian}}$ cluster (see also Fig S5), dispersion pattern of the virus between Afghanistan and Iran, the evolutionary history of the North American CRF35_AD-like strain, and the linkage between CRF35_AD strains identified among Afghan refugees and the CRF35_AD epidemic in Afghanistan and Iran. Similar to the complete analyses, we could not clearly identify the country (i.e., Afghanistan or Iran) that first established or received the CRF35_AD epidemic. In the *gag*_2 region, the total number of sequences available from Iran was small (n=21); therefore, only two balanced subsets were created and analyzed for this region. For each genomic region, the color code is indicated in the legend, on the lower left corner. To the upper left of each phylogeny, probable ancestral locations with their posterior probabilities are indicated for the CRF35_A$_{\text{Afghan-Iranian}}$ cluster (Node A). Posterior clade credibility values greater than 0.7 are indicated for the key nodes. Parental sequences are collapsed for visual clarity. **PSP:** Posterior State Probability; **A1:** HIV-1 subtype A1; **D:** HIV-1 subtype D; **AF:** Afghanistan; **IR:** Iran; **AF refugees $_{\text{PK}}$:** Afghan refugees living in Pakistan
