# Supplementary material for: Spatio-Temporal History of HIV-1 CRF35_AD in Afghanistan and Iran
Source: PLoS One. 2016 Jun 9;11(6):e0156499. doi: 10.1371/journal.pone.0156499 (PMC4900578; doi:10.1371/journal.pone.0156499)
Supplement: S1 Table — (PDF) [file pone.0156499.s008.pdf]

S1 Table. HIV-1 CRF35_AD datasets used in this study

		Genomic Region			
**Country**	**City (direction)**	***gag*_1**	***gag*_2**	***pol*_1***	***pol*_2**
**Afghanistan**	Herat (W)	9	9	9	9
	Kabul (E)	4	4	4	4
**Pakistan§**	Karachi (S)	-	3	-	-
**Iran**	Mashhad (NE)	-	11	-	18
	Borujerd (W)	-	-	-	16
	Sanandj (W)	-	-	35	35 (6)
	Kermanshah (W)	9	3	9	9
	Shiraz (S)	9	3	9	9
	Tehran (C)	33	4	49 (4)	169
**USA**	Virginia State	-	-	-	1
**Total**		64	37	115	270
**Sampling interval**		9 (2002-2011)	6 (2005-2011)	7 (2005-2012)	7 (2005-2012)

* Only this dataset encompassed the genomic region of CRF35_AD that belonged to the "D" parent of CRF35_AD. The number in the parentheses refers to the number of sequences excluded from the analyses, due to drug resistance mutation. § CRF35_AD samples from Pakistan belong to Afghan refugees living in Pakistan. **N:** North; **S:** South; **E:** East; **W:** West; **C:** Center
