# Supplementary material for: Spatio-Temporal History of HIV-1 CRF35_AD in Afghanistan and Iran
Source: PLoS One. 2016 Jun 9;11(6):e0156499. doi: 10.1371/journal.pone.0156499 (PMC4900578; doi:10.1371/journal.pone.0156499)
Supplement: S2 Table — (PDF) [file pone.0156499.s009.pdf]

S2 Table. HIV-1 parental A1 and D sequences used in Bayesian phylogeographic analyses

Region	Country	Genomic Region			
		***gag*_1**	***gag*_2**	***pol*_1***	***pol*_2**
Asia	Afghanistan†	-	7	-	-
	Pakistan	-	9	-	-
Africa§	Kenya	100	22	64 (5)	61 (1)
	Uganda	45	25	77 (2)	38
**Total**		145	63	141	99
**Sampling interval**		26 (1985-2011)	26 (1985-2011)	22 (1990-2012)	29 (1985-2014)

* Only this dataset belonged to the "D" parent. The number in the parentheses refers to the number of sequences excluded from the analyses, due to drug resistance mutation. † Refers to Afghan refugees living in Pakistan. § A subset of non-redundant sequences was selected for Kenya and Uganda. To do so, highly similar (identity ≥ 90%) sequences from each country were clustered with the CD-HIT program [1], using an online web server [2], and only one sequence per cluster was selected.

References

1. Li W, Godzik A. Cd-hit: a fast program for clustering and comparing large sets of protein or nucleotide sequences. Bioinformatics. 2006 22: 1658–59.

2. Huang Y, Niu B, Gao Y, Fu L, Li W. CD-HIT Suite: a web server for clustering and comparing biological sequences. Bioinformatics. 2010 26: 680–82.
