# Supplementary material for: Spatio-Temporal History of HIV-1 CRF35_AD in Afghanistan and Iran
Source: PLoS One. 2016 Jun 9;11(6):e0156499. doi: 10.1371/journal.pone.0156499 (PMC4900578; doi:10.1371/journal.pone.0156499)
Supplement: S3 Table — (PDF) [file pone.0156499.s010.pdf]

S3 Table. Complete and country-balanced CRF35_AD datasets used for sensitivity analyses

Dataset	Country	Genomic Region			
		*gag*_1	*gag*_2†	*pol*_1*	*pol*_2
**Complete**	**Afghanistan**	13	16	13	13
	**Iran**	51	21	102	256
	**USA**	-	-	-	1
**Subset 1**	**Afghanistan**	13	16	13	13
	**Iran**	13	13	13	19
	**USA**	-	-	-	1
**Subset 2**	**Afghanistan**	13	16	13	13
	**Iran**	12	13	13	17
	**USA**	-	-	-	1
**Subset 3**	**Afghanistan**	13	-	13	13
	**Iran**	13	-	13	17
	**USA**	-	-	-	1
**Subset 4**	**Afghanistan**	13	-	13	13
	**Iran**	13	-	13	17
	**USA**	-	-	-	1

†At this genomic region, the total number of sequences available from Iran was small (n=21); therefore, only two balanced subsets were created. * Only this dataset belonged to the "D" parent.
